# Reflex Testing for Hepatitis D Infection: A Unique Opportunity to Reduce Hepatitis D‐Related Chronic Liver Disease Deaths in Australia

**DOI:** 10.5694/mja2.70170

**Published:** 2026-04-05

**Authors:** Jessica Howell, Lauren Andersson, Miriam T. Levy, James O'Beirne, Leon Adams, Katharine Irvine, Avik Majumdar, Golo Ahlenstiel, Kathy Jackson, Krispin Hajkowicz, Joseph Doyle, Jane Davies, Sarah Cherian, Wayne Dimech, Alexander J. Thompson

**Affiliations:** ^1^ St Vincent's Hospital Melbourne Melbourne Victoria Australia; ^2^ Burnet Institute Melbourne Victoria Australia; ^3^ University of Melbourne Melbourne Victoria Australia; ^4^ Monash University Melbourne Victoria Australia; ^5^ Peter MacCallum Cancer Institute Melbourne Victoria Australia; ^6^ Liverpool Hospital Sydney Sydney New South Wales Australia; ^7^ Sunshine Coast University Hospital Sunshine Coast Queensland Australia; ^8^ University of Western Australia Perth Western Australia Australia; ^9^ Sir Charles Gairdner Hospital Perth Western Australia Australia; ^10^ University of Queensland Brisbane Queensland Australia; ^11^ AW Morrow Gastroenterology and Liver Centre Royal Prince Albert Hospital Sydney New South Wales Australia; ^12^ Austin Hospital Melbourne Victoria Australia; ^13^ Western Sydney University Sydney New South Wales Australia; ^14^ Blacktown and Mount Druitt Hospitals Western Sydney Local Health District Sydney New South Wales Australia; ^15^ Victorian Infectious Diseases Reference Laboratory Doherty Institute Melbourne Victoria Australia; ^16^ Alfred Health Melbourne Victoria Australia; ^17^ Menzies School of Health Research Darwin Northern Territory Australia; ^18^ Royal Darwin Hospital Darwin Northern Territory Australia; ^19^ Sullivan Nicolaides Pathology Brisbane Queensland Australia; ^20^ National Research Laboratory Melbourne Victoria Australia

**Keywords:** clinical pathology, hepatitis B, hepatocellular carcinoma, liver cirrhosis, liver diseases, viral hepatitis

## Abstract

Chronic hepatitis D virus (HDV) infection always occurs as a coinfection with hepatitis B virus (HBV) and is the most severe form of viral hepatitis, associated with a high risk of cirrhosis, liver cancer and death. Effective treatment is now available for HDV–HBV coinfection and HDV screening is recommended for all people living with HBV, yet most people in Australia with HDV–HBV are diagnosed too late to prevent complications. This article calls for an urgent change in HDV testing policy and funding to implement reflex HDV antibody (anti‐HDV) testing for all people diagnosed with HBV infection, thus enabling timely diagnosis of HDV–HBV coinfection and rapid access to life‐saving treatment.

## 1 Introduction

Chronic hepatitis D virus (HDV) infection always occurs as a coinfection with hepatitis B virus (HBV) and is the most severe form of viral hepatitis [1, 2]. Over 25 million people worldwide are estimated to be living with HDV–HBV coinfection and are at risk of death from cirrhosis and liver cancer [1, 3]. Due to the severity of liver disease associated with HDV–HBV coinfection, Australian and international clinical guidelines recommend HDV screening with a HDV antibody (anti‐HDV) test for all people with HBV infection [1, 4]. A new treatment for HDV infection is available [5, 6, 7, 8]; however, most people do not receive life‐saving treatment early enough to prevent cirrhosis and liver cancer [1]. This is because hepatitis D awareness and testing rates among people with hepatitis B are unacceptably low in Australia [9, 10, 11].

The Australian Government is introducing universal HBV testing for all Australians born before 2000, in line with the new National Hepatitis B Strategy 2025–2030 recommendations [12]. We should harness this unique opportunity to implement new approaches to HDV testing now to ensure people with HBV–HDV coinfection receive timely HDV diagnosis and treatment to prevent deaths.

As representatives of the Gastroenterological Society of Australia (GESA) and Hepatitis Australia and as experts in hepatology, infectious diseases and pathology, we call for a change in HDV testing policy and funding to implement reflex anti‐HDV antibody testing for all people diagnosed with HBV infection.

## 2 Epidemiology and Disease Burden From Chronic Hepatitis B–D Virus Coinfection

The global distribution of HDV is heterogenous, with prevalence estimates for HDV–HBV coinfection of 5%–13% of people with chronic HBV infection [1, 2, 3]. Pakistan, India, Mongolia, Central Asia, Eastern Europe, sub‐Saharan Africa, the Amazon Basin and Pacific Islands and Territories below the equator such as Kiribati have the highest HDV–HBV coinfection prevalence [2, 3]. In these regions, prevalence of HDV is 10%–70% among people with chronic HBV infection [3]. The burden of HDV–HBV coinfection in Australia is poorly understood, in part due to low HDV testing rates [9, 10, 11]. HDV–HBV coinfection prevalence is affected by changing migration patterns from countries with high prevalence [1]. In a retrospective cohort study from Queensland, 179 individuals were diagnosed with HDV–HBV coinfection between 1997 and 2016, with 4.1% seroprevalence among those tested [9]. In Victoria, 190 people tested anti‐HDV antibody positive between 2010 and 2016, of whom 87% were HDV RNA positive, consistent with active infection [10]. In a recent study from pathology services across Queensland and Victoria conducted between 2018 and 2022, 42% of people with HBV infection were screened for HDV, of whom 1.3%–3.8% were anti‐HDV positive and 34%–67% were HDV RNA positive [11]. These data suggest that about 1%–3% of estimated people living with hepatitis B in Australia (219,800) may have active HDV infection and require treatment (2198–6594 individuals) [11, 13].

HDV is transmitted parenterally, either as a coinfection with HBV or as a super‐infection in someone with HBV, using the same hepatocyte sodium taurocholate co‐transporting polypeptide (NTCP) entry receptor as HBV [1]. In contrast to other hepatitis viruses, HDV is a defective RNA virus that can only survive in the hepatocytes of people with HBV infection, as it uses HBV viral machinery for cell entry and reproduction [1]. Risk factors for transmission of HDV infection are similar to other bloodborne viruses, including unsafe injections and medical procedures, sharing of injecting equipment and other blood exposures [1, 2].

Acute coinfection with HBV–HDV leads to acute hepatitis of varying severity, from asymptomatic to fulminant liver failure [1, 2]. Super‐infection with HDV more commonly leads to chronic HDV–HBV infection, which is associated with more rapid liver disease progression and two to three times higher rates of cirrhosis and hepatocellular carcinoma than in chronic HBV mono‐infection [1]. In an Australian study, people with HDV–HBV coinfection were twice as likely to have cirrhosis and almost twice as likely to require liver transplantation compared to those with chronic HBV infection alone [8]. Fulminant hepatitis may also occur uncommonly [1].

## 3 Diagnosis of HDV Infection

HDV exposure is confirmed by the presence of anti‐HDV antibody. However, anti‐HDV antibody may persist for years after declines in HDV RNA level, even after hepatitis B surface antigen (HBsAg) seroconversion is achieved. Therefore, to diagnose active HDV infection, a HDV RNA polymerase chain reaction (PCR) test is required [1]. Currently, Australian and international HBV guidelines recommend anti‐HDV antibody testing for all people with chronic HBV and subsequent HDV RNA PCR testing for anti‐HDV‐positive individuals to confirm active infection [1, 4]. Anti‐HDV antibody testing is covered by Medicare, but uptake remains low in Australia [9, 10, 11]. HDV RNA PCR testing is not currently covered by Medicare.

## 4 Treatment of HDV Infection

The mainstay of therapy for HBV–HDV coinfection has been nucleos(t)ide analogue suppressive therapy for HBV and pegylated interferon for HDV. However, only 17%–30% of people achieve viral suppression after 24–48 weeks of pegylated interferon therapy, over 50% relapse with treatment cessation and tolerability is poor [1, 5]. Bulevirtide, an NTCP entry receptor inhibitor that blocks both HDV and HBV entry into hepatocytes, is a new HDV treatment that can be used with pegylated interferon or alone. In a phase 2B study (*n* = 174) of 10 mg bulevirtide with pegylated interferon α‐2a, 46% achieved undetectable HDV RNA and over half achieved alanine aminotransferase (ALT) normalisation after 24 weeks, compared to 17% with pegylated interferon alone [5]. A phase 2B open‐label study of bulevirtide monotherapy with tenofovir (*n* = 120), including 59 people with cirrhosis, showed 53% of people receiving 2 mg daily bulevirtide achieved undetectable HDV RNA at 24 weeks and 27% cleared HBsAg, with excellent tolerability (3% serious adverse events) [6]. A phase 3 randomised trial of bulevirtide monotherapy (*n* = 150) confirmed these findings: 76% achieved > 2 log drop in HDV viral load and 16% achieved HDV clearance at 48 weeks, with no serious adverse events [7]. Bulevirtide has comparable response rates to pegylated interferon monotherapy with higher tolerability and can be used in people for whom pegylated interferon is contraindicated.

Critically, emerging data from a retrospective study suggest that bulevirtide can be used (off‐label) safely without pegylated interferon in people with advanced HDV cirrhosis—a group that historically could not be treated—to restore liver function and prevent life‐threatening liver decompensation [8]. In 19 people with decompensated cirrhosis, 74% achieved > 2 log drop in viral load, 74% achieved ALT normalisation and 47% recompensated from Child–Pugh B to A stage cirrhosis [7]. Although not yet available on the Pharmaceutical Benefits Scheme, bulevirtide is approved for use in Australia. Early diagnosis of HDV–HBV coinfection is vital to ensure early treatment to prevent progression to cirrhosis, liver decompensation and hepatocellular carcinoma.

## 5 Optimising the Diagnosis of HDV Infection

Introduction of universal testing for chronic HBV in Australia represents a time‐critical opportunity to implement routine HDV screening among people with chronic HBV infection. HDV testing is recommended in Australian and international guidelines for all people living with HBV [1, 4]. Universal HDV screening at the time of HBV diagnosis enables rapid early referral to specialist hepatology services for cirrhosis evaluation and management, HDV treatment and enrolment in liver cancer surveillance. This is especially important in the face of the necessary shift towards community‐based care for chronic HBV, to ensure that people with HDV–HBV coinfection who are at greater risk of rapid disease progression and death are appropriately referred for early hepatology care. Blood‐based biomarkers and transient elastography are not as reliable for diagnosis of cirrhosis in people with HDV–HBV coinfection; therefore, liver biopsy is required to confirm cirrhosis diagnosis [1]. However, as HDV–HBV coinfection is uncommon, awareness among affected communities and demand for testing are low [1].

**FIGURE 1:**
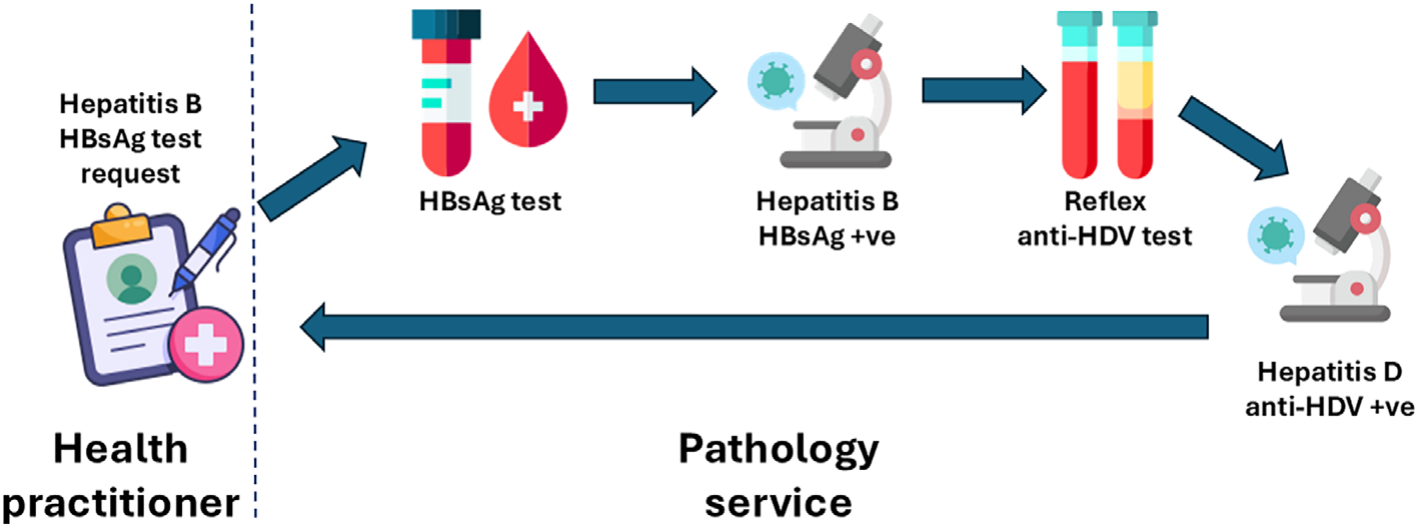
Process for reflex hepatitis D virus antibody (anti‐HDV antibody) testing. Health worker orders a hepatitis B surface antigen (HBsAg) test. At the laboratory, if the HBsAg test is positive indicating active hepatitis B infection, an anti‐HDV antibody test is automatically ordered using the same blood sample, without the health worker needing to order it. The results of the HBsAg test and anti‐HDV antibody test are then sent to the ordering health worker.

Reflex, or automatic, testing for HDV infection in people who are diagnosed with HBV infection by pathology services, without the need for a health worker to request the anti‐HDV test (Figure 1), would substantially simplify HDV screening among people with chronic HBV infection. Reflex anti‐HDV antibody testing when an individual tests positive for HBsAg avoids the need for a client to return to their treating doctor, receive a new request slip for anti‐HDV antibody test, attend their pathology service again and attend a further appointment with their treating doctor for the result. This multi‐step process is inefficient and costly and leads to attrition of people at each step of the diagnostic process. It also relies on health workers to recognise the need for anti‐HDV screening among people with chronic HBV infection. The policy shift to universal HBV testing recognises the limitations of a risk‐based strategy. Similarly, data show that risk‐based testing for HDV–HBV coinfection misses a substantial proportion of those with HDV infection and testing rates are low even where universal testing is recommended [14, 15]. A recent study showed a fivefold increase in HDV diagnosis when reflex HDV testing for people diagnosed with HBV was introduced—two‐thirds of whom would not have been identified by risk‐based testing and two‐thirds already had cirrhosis [15]. Local data from a pilot study in Queensland showed reflex testing led to a 28% increase in HDV screening uptake among people with HBV [11]. Several centres around the world have adopted reflex anti‐HDV antibody testing based on these compelling data [1, 15], and reflex anti‐HDV antibody testing is now recommended in the World Health Organization 2024 HBV guidelines [16].

There is a strong precedent for reflex testing by pathology services for viral hepatitis in Australia. In recognition of the attrition of people from the hepatitis C cascade of care and proven cost‐effectiveness of reflex hepatitis C virus antibody (anti‐HCV) testing in Australia [17, 18], reflex HCV RNA testing can now be performed by laboratories where an anti‐HCV antibody test is positive or indeterminate and this is covered by Medicare [17]. We advocate for a similar approach to screening for HDV: health policy, systems and Medicare rebates should change to enable pathology services to reflex test for anti‐HDV antibody in people who test positive for HBsAg, which could use the same blood sample to minimise costs. Strategies to reduce duplicate anti‐HDV testing should also be explored to improve cost‐effectiveness of reflex anti‐HDV testing through enhanced data sharing across health services. Consent for HDV testing should be provided at the time of counselling for HBV testing by their health worker. HDV is a notifiable illness; therefore, reflex testing supports public health networks in case finding to ensure that people with HDV–HBV coinfection are linked to timely care and treatment to reduce deaths. Although increasing global hepatitis B vaccination coverage [3] coupled with emerging hepatitis B curative regimens may reduce HDV–HBV coinfection incidence and prevalence in the future, screening for HDV infection in people with hepatitis B will remain a critical step to prevent deaths. Further studies are required to confirm the cost‐effectiveness of HDV reflex testing in the Australian context. Moreover, although HDV RNA PCR testing is more expensive than anti‐HDV antibody testing, a similar case can be made in support of reflex HDV RNA testing among those who test anti‐HDV antibody positive to improve timely diagnosis of active HDV–HBV coinfection. This also warrants evaluation in cost‐effectiveness studies.

## 6 Conclusion

HDV–HBV coinfection is uncommon in Australia, yet carries a high mortality risk from cirrhosis and hepatocellular carcinoma. Improving awareness and enabling reflex anti‐HDV antibody testing through health policy change are two ways we can improve HDV–HBV diagnosis, linkage to specialist hepatology care and timely treatment to reduce the health burden of this uncommon but devastating liver disease in Australia.

## Author Contributions

**Jessica Howell:** conceptualisation, writing (original draft). **Lauren Andersson:** writing (original draft). **Miriam T. Levy:** writing (reviewing and editing). **James O'Beirne:** writing (reviewing and editing). **Katharine Irvine:** writing (reviewing and editing). **Leon Adams:** writing (reviewing and editing). **Golo Ahlenstiel:** writing (reviewing and editing). **Avik Majumdar:** writing (reviewing and editing). **Joseph Doyle:** writing (reviewing and editing). **Jane Davies:** writing (reviewing and editing). **Sarah Cherian:** writing (reviewing and editing). **Wayne Dimech:** writing (reviewing and editing). **Alexander J. Thompson:** writing (reviewing and editing). **Kathy Jackson:** writing (reviewing and editing). **Krispin Hajkowicz:** writing (reviewing and editing).

## Funding

The authors have nothing to report.

## Disclosure

Not commissioned; externally peer reviewed.

## Conflicts of Interest

Several authors have received funding from Gilead (which manufactures and sells bulevirtide) unrelated to this article. There was no industry involvement in the conceptualisation, design, writing or editing of this manuscript. This work was initiated and conducted solely by the Gastroenterological Society of Australia (GESA) Liver Faculty, with invited leading researchers and professional and community organisation stakeholders in the field. Jessica Howell has received investigator‐initiated funding (2021) from Gilead Sciences, speaker fees from Roche Pharmaceuticals (2024) and participated in Advisory boards for Roche Diagnostics and Astra Zeneca (2024), all unrelated to the current work. Lauren Andersson has received a Gilead Australia Fellowship for an investigator‐initiated project in hepatitis D epidemiology in Victoria, unrelated to the current work. Krispin Hajkowicz has received investigator‐initiated funding from Gilead Sciences unrelated to the current work. Kathy Jackson has received investigator‐initiated funding from Gilead Sciences, unrelated to the current work. Miriam T. Levy has had consulting/advisory roles for Gilead Sciences and AstraZeneca; research funding from Gilead Sciences and AbbVie, and travel sponsorship from IPSEN all unrelated to the current work. James O'Beirne has had consulting/advisory roles with CSL, Roche diagnostics, Norgine and Novo‐Nordisk, and has received a travel grant from IPSEN pharmaceuticals, all unrelated to the current work. Leon Adams has received grant funding and speaker fees from Novo Nordisk, speaker fees from Falk Pharma and participated in advisory boards for Novo Nordisk, CSL Behring, all unrelated to the current work. Joseph Doyle's institution has received funding for research and consulting from Gilead Sciences and Abbvie, all unrelated to the current work. Alexander J. Thompson has received speaker fees from Ipsen, Abbvie, Gilead Sciences and Roche Diagnostics, investigator‐initiated grant support from Gilead Sciences, Abbvie and Roche Diagnostics, and has been on advisory boards for Gilead Sciences, Abbvie, Ipsen, Roche Diagnostics, Roche and BMS, all unrelated to the current work. Jessica Howell, James O'Beirne, Miriam T. Levy, Katharine Irvine, Golo Ahlenstiel, Leon Adams and Avik Majumdar are all members of the GESA Liver Faculty as outlined in the manuscript. Joseph Doyle is President of Hepatitis Australia. Jane Davies is Chair of Viral Hepatitis Faculty, Australasian Society for Infectious Diseases. All other authors declare that they have no conflicts of interest. The opinions here are personal and do not reflect those of employers, associations or government.

## Data Availability

The authors have nothing to report.
